# Effects of Molecular Weight of Functionalized Liquid Butadiene Rubber as a Processing Aid on the Properties of SSBR/Silica Compounds

**DOI:** 10.3390/polym13060850

**Published:** 2021-03-10

**Authors:** Donghyuk Kim, Byungkyu Ahn, Kihyun Kim, JongYeop Lee, Il Jin Kim, Wonho Kim

**Affiliations:** 1 School of Chemical Engineering, Pusan National University, Busan 46241, Korea; ehdgurzxc@gmail.com; 2 Hankook Tire & Technology Co., Ltd., R&D Center, 50 Yuseong-daero 935beon-gil, Yuseong-gu, Daejeon 34127, Korea; bkahn8855@gmail.com (B.A.); k.kim@hankooktire.com (K.K.); jylee@hankooktire.com (J.L.); 3 Hankook Tire & Technology Co., Ltd., 286 Pangyo-ro, Bundang-gu, Seongnam-si, Gyeonggi-do 13494, Korea; tigerkim@hankooktech.com

**Keywords:** SSBR, liquid butadiene rubber, silica-filled compound, anionic polymerization, rubber compounding

## Abstract

Liquid butadiene rubber (LqBR) which used as a processing aid play a vital role in the manufacturing of high-performance tire tread compounds. However, the studies on the effect of molecular weight, microstructure, and functionalization of LqBR on the properties of compounds are still insufficient. In this study, non-functionalized and center-functionalized liquid butadiene rubbers (N-LqBR and C-LqBR modified with ethoxysilyl group, respectively) were synthesized with low vinyl content and different molecular weights using anionic polymerization. In addition, LqBR was added to the silica-filled SSBR compounds as an alternative to treated distillate aromatic extract (TDAE) oil, and the effect of molecular weight and functionalization on the properties of the silica-filled SSBR compound was examined. C-LqBR showed a low Payne effect and Mooney viscosity because of improved silica dispersion due to the ethoxysilyl functional group. Furthermore, C-LqBR showed an increased crosslink density, improved mechanical properties, and reduced organic matter extraction compared to the N-LqBR compound. LqBR reduced the glass transition temperature (T_g_) of the compound significantly, thereby improving snow traction and abrasion resistance compared to TDAE oil. Furthermore, the energy loss characteristics revealed that the hysteresis loss attributable to the free chain ends of LqBR was dominant.

## 1. Introduction

Liquid butadiene rubber (LqBR) is widely used as a vulcanizable plasticizer in the fields of plastics, tire, printing inks, paints, coatings, and sealants, etc. [1]. Low molecular weight LqBR generally has a polydiene structure with a molecular weight (1000–50,000 g/mol) between that of a solid polymer and processing aid, which is synthesized by a method similar to that of solid polymers or co-polymers [2,3]. The first commercial LqBR “Plastikator 32” was produced in Germany in 1925, and the major polymer companies (Dupont of Delaware in USA, Colorado Chemicals of New Jersey in USA, and Ricon of California in USA) began to develop the production technology of LqBR and conduct research on its application between 1950 and mid-1960s [4]. The market size increased rapidly as LqBR was used as a raw material for coatings and inks in Western Europe and Japan since 1970. Interests in LqBR have increased significantly since 1990 as it has been used to serve the purpose of plasticizers, cure coagents, or to improve the viscoelastic properties of tire treads in the rubber industry. In particular, the number of studies on its application in the tire industry has increased significantly over the past decade, as indicated by a significant increase in the number of patents related to liquid polymers [5].

Since 1995, European processing oil producers and tire manufacturers have begun to develop processing aids to replace highly aromatic oils (distilled aromatic extracts, DAEs) that contain polycyclic aromatic hydrocarbons (PAHs), which are carcinogenic substances. In fact, the use of DAE oils is prohibited in tire manufacturing, and oils with a low PAH content, such as treated distillate aromatic extract (TDAE) oil, are being used as alternative processing aids in accordance with European Registration, Evaluation, Authorization and Restriction of Chemicals (EU REACH) regulations in 2010 [6,7]. However, TDAE oil-applied vulcanizates have a disadvantage in that compound properties are degraded over time due to the migration of TDAE oil [8,9]. These challenges suggested the need for a new, non-PAH processing aid with low glass transition temperature (T_g_) and no migration. Accordingly, the demand and interest in LqBR began to increase gradually.

The technological applications advanced gradually with various attempts to use LqBR as a processing aid in the manufacturing of car tires. In particular, Kuraray Co. Ltd. of Tokyo in Japan significantly reduced T_g_ by minimizing the vinyl content of the non-functionalized LqBR, which was found to have reduced the migration of LqBR through co-vulcanization with a solid polymer [10]. Sumitomo Rubber Industries and Continental AG improved abrasion resistance and viscoelastic performance at low temperatures by applying LqBR with low vinyl content to winter tire compounds [11,12]. Hirata et al. applied commercialized non-functionalized liquid rubbers as processing aids to carbon black-filled NR compounds, thereby overcoming the processability and migration problems [13]. In contrast, Choi et al. applied commercialized LqBR to a silica-filled styrene-butadiene rubber (SBR) compound, which was found to have improved processability and reduced the modulus due to the decrease in Mooney viscosity and the decrease in crosslink density, respectively. In addition, it was shown that the application of LqBR that was modified with maleic anhydride improved silica dispersion in comparison with the application of non-functionalized LqBR; this is because a chemical bond with the silanol group on the silica surface could be formed in the case of the former [14]. Additional modifications of LqBR with alkoxy groups, amino groups, cyano groups, sulfonyl groups, epoxy groups, and halogen atoms further improved as it can form chemical bonds with silanol groups present on the silica surface [15,16,17].

However, most previous studies used commercialized LqBR instead of in house synthesized LqBR, so the structure of LqBR was limited with respect to control of molecular weight, vinyl content, and functionalization. Due to this limitation, the underlying mechanism of the functions of LqBR in the compound and the resulting properties could not be explained. Moreover, the studies on the effect of molecular weight, microstructure, and functionalization of LqBR on the properties of compounds are still insufficient.

Therefore, center-functionalized LqBR (C-LqBR, modified with ethoxysilyl group) and non-functionalized LqBR (N-LqBR) with low vinyl content and different molecular weights were synthesized using anionic polymerization in this study. In addition, silica-filled SSBR compounds were prepared by applying LqBR as a processing aid to replace TDAE oil, and the effects of the molecular weight and functionalization of LqBR were examined by evaluating the properties that affect the performance of the tire tread compound. The properties were compared after controlling the content of sulfur and accelerator to have a crosslink density similar to that of the compound to which only TDAE oil was applied. The results presented and analyzed in this study are expected to provide the basis for designing and selecting the optimal LqBR required for manufacturing tire tread compounds to which LqBR is applied.

## 2. Materials and Methods

### 2.1. Materials

#### 2.1.1. Polymerization

All materials used in the polymerization were purged with nitrogen, while cyclohexane (99%, Samchun Chemical Co., Seoul, Korea) was used as an organic solvent and n-butyl lithium was used as an initiator for anionic polymerization. 1,3-Butadiene (Kumho Petrochemical Co., Daejeon, Korea) was used as a monomer, and anisole (99%, Samchun Chemical Co., Seoul, Korea) was used as a polar modifier to control the vinyl content. Tetraethyl orthosilicate (TEOS, 98%, Sigma-Aldrich Corp., Seoul, Korea) was used as a coupling agent, and benzyl alcohol (99%, Samchun Chemical Co., Seoul, Korea) was used as a terminating agent.

#### 2.1.2. Compounding

SSBR (SOL-5220M, Kumho Petrochemical Co. Daejeon, Korea, styrene content: 26.5 wt%, vinyl content: 26 wt%, non-oil extended) was used as the base polymer. Silica (Ultrasil 7000 GR, Evonik Industries AG, Essen, Germany, Brunauer–Emmett–Teller (BET) surface area: 160–175 m^2^/g) was used as a filler; and bis-[3-(triethoxysilyl)propyl]tetrasulfide (TESPT, Si-69, Evonik Korea Ltd., Seoul, Korea) was used as a silane coupling agent. Moreover, TDAE oil (Kukdong Oil & Chemicals Co., Yangsan, Korea) was used as a processing aid for mixing. ZnO and stearic acid (both from Sigma-Aldrich Corp., Seoul, Korea) were used as activators, and N-(1,3-dimethylbutyl)-N-phenyl-p-phenylenediamine (6PPD, Kumho Petrochemical Co., Daejeon, Korea) was used as an antioxidant in the compound. Additionally, sulfur (Daejung Chemicals & Metals Co., Siheung, Korea) was used as a crosslinking agent. N-cyclohexyl benzothiazole-2-sulfenamide (CBS, 98%, Tokyo Chemical Industry Co. Ltd., Tokyo, Japan) and 1,3-diphenylguanidine (DPG, 98%, Tokyo Chemical Industry Co. Ltd., Tokyo, Japan) were used as cure accelerators.

### 2.2. Measurements

#### 2.2.1. Gel Permeation Chromatography

Molecular weight and molecular weight distribution were measured using gel permeation chromatography (GPC) system (Shimadzu, Kyoto, Japan). The GPC system consisted of a solvent delivery unit, reflective index detector, and three types of Styragel columns: HT 6E (10 µm, 7.8 mm × 300 mm), HMW 7 column (15–20 µm, 7.8 mm × 300 mm), and HMW 6E column (15–20 µm 7.8 mm × 300 mm). Further, the measured molecular weight was corrected using a polystyrene standard sample (EasiCal PS-1 standard, Agilent Technologies, Santa Clara, CA, USA).

#### 2.2.2. Proton Nuclear Magnetic Resonance Spectroscopy (^1^H NMR)

The vinyl content in LqBR was evaluated using proton nuclear magnetic resonance spectroscopy (^1^H NMR; Varian, Unity Plus 300 spectrometer, Garden State Scientific, Morristown, NJ, USA). LqBRs were dissolved in a 5 mm NMR tube at a concentration of 15 mg/mL using deuterochloroform (CDCl_3_, Cambridge Isotope Laboratories, Inc., Andover, MA, USA) as a solvent.

#### 2.2.3. Differential Scanning Calorimetry (DSC)

The determination of glass-transition temperature (T_g_) was carried out on a differential scanning calorimeter (DSC-Q10, TA Instruments, Delaware, USA). The curves for samples (3–6 mg) were obtained by heating sample from −120 to −20 °C at a heating rate of 10 °C/min under nitrogen atmosphere.

#### 2.2.4. Payne Effect

A rubber processing analyzer (RPA2000, Alpha Technologies, Hudson, OH, USA) was used to evaluate the silica dispersion and filler–filler interaction of the compound according to ASTM D8059. The storage modulus (G′) of the uncured compound was measured at a temperature of 60 °C in the range of 0.28%–40.04% strain. The G′ value is large in the low-strain region because the silica agglomerates are not destroyed, whereas the G′ value decreased in the high-strain region as the agglomerates are destroyed. Therefore, the change in G′, i.e., △G′ (G′ at 0.28% − G′ at 40.04%) is called the Payne effect, which indicates the degree of filler–filler interaction.

#### 2.2.5. Mooney Viscosity

A Mooney viscometer (Vluchem IND Co., Seoul, Korea), which evaluates the processability of rubber by measuring the torque when the rotor rotates in a space filled with uncured rubber compound according to ASTM D1646, was used. The rotation speed of the rotor was set to 2 rpm, and the temperature was set to 100 °C. The torque value was measured by rotating the rotor for 4 min after preheating for 1 min.

#### 2.2.6. Cure Characteristics

For measuring the cure characteristics of the compound, a moving die rheometer (MDR, Myung Ji Co., Seoul, Korea) was operated for 30 min, maintaining a vibration angle of ±1° and temperature of 160 °C while the minimum torque (T_min_), maximum torque (T_max_), scorch time (t_10_), and optimal cure time (t_90_) were measured.

#### 2.2.7. Solvent Extraction and Crosslink Density

The crosslink density is defined as the number of crosslink points in the vulcanizates. The higher crosslinking density means that the molecular weight between the crosslink points decreases, and the number of crosslink points increases. In the swelling tests, the higher the crosslinking density, the less the number of solvent molecules can permeate between the crosslinked rubber chains, and thus relatively less swelling. The vulcanizates with dimensions of 10 mm × 10 mm × 2 mm were weighed and immersed in tetrahydrofuran (THF, 99%, Samchun Chemical Co., Seoul, Korea) and n-hexane (95%, Samchun Chemical Co., Seoul, Korea) at 25 °C for 2 days to remove organic additives inside the sample. Subsequently, the weight was measured after the sample was dried at 25 °C for 1 day to determine the mass fraction of the extracted organic additive. The weight of the sample immersed and swollen in a toluene solvent for 1 day at room temperature was measured after measuring the weight of the dry sample in order to calculate the total crosslink density. Moreover, the total crosslink density and average molecular weight between the crosslink points, *M_c_*, were calculated using the Flory–Rehner equation [18,19,20,21].

(1) $$\nu =\frac{1}{2M_{c}}=-\frac{\ln\left(1-v_{r}\right)+v_{r}+\chi v_{r}^{2}}{2\rho V_{s}\left(v_{r}^{\frac{1}{3}}-v_{r}/2\right)},$$

where *ν* is the crosslink density (mol/g), *M_c_* is the average molecular weight between crosslink points (g/mol), *ν_r_* is the volume fraction of rubber in the swollen gel at equilibrium in Equation (2), *V_s_* is the molar volume of solvent (cm^3^/mol), ρ is the density of rubber sample (g/cm^3^), and χ is the polymer–solvent interaction parameter in Equation (3).

(2) $$v_{r}=\frac{\frac{w_{dry}-w_{filler}}{\rho_{rubber}}}{\begin{array}{c} \frac{w_{dry}-w_{filler}}{\rho_{rubber}}+\frac{w_{swollen}-w_{dry}}{\rho_{solvent}} \end{array}},$$

where *w_dry_* is the weight of dry sample, *w_filler_* is the weight of filler in the dry sample, *w_swollen_* is the weight of the swollen sample, *ρ_rubber_* is the density of the rubber, and *ρ_solvent_* is the density of the solvent.

(3) $$\chi =0.34+\frac{v_{0}}{\begin{array}{c} RT \end{array}}{\left(\delta_{p}-\delta_{s}\right)}^{2}$$

where *ν*_0_ is the molar volume of solvent, *ẟ_p_* is the solubility parameter of polymer, *ẟ_s_* is the solubility parameter of solvent.

#### 2.2.8. Mechanical Properties

A 100 mm (length) × 25 mm (width) dumbbell-shaped specimen that was prepared according to ATSM D 412 was measured at a speed of 500 mm/min using a universal testing machine (UTM, KSU-05M-C, KSU Co., Ansan, Korea) to measure the mechanical properties (tensile strength, modulus, and elongation at break) of vulcanizates.

#### 2.2.9. Abrasion Resistance

The abrasion resistance was measured according to DIN 53516 using a Deutsche Industrie Normen (DIN) abrasion tester. The specimen was prepared in a cylindrical shape with a diameter of 16 mm and thickness of 8 mm. The mass reduction after moving the specimen 40 m across the surface of an abrasive sheet mounted on a cylindrical drum revolving at 40 ± 1 rpm was measured by applying a load of 5 N.

#### 2.2.10. Viscoelastic Properties

For the dynamic viscoelastic properties of the compound, the storage modulus (G′), loss modulus (G″), and tan ẟ were measured from −60 to 70 °C under a 10 Hz frequency at 0.5% strain using a strain-controlled rheometer (ARES-G2, TA Instrument).

### 2.3. Synthesis and Functionalization of Liquid Butadiene Rubbers

**Scheme 1:**
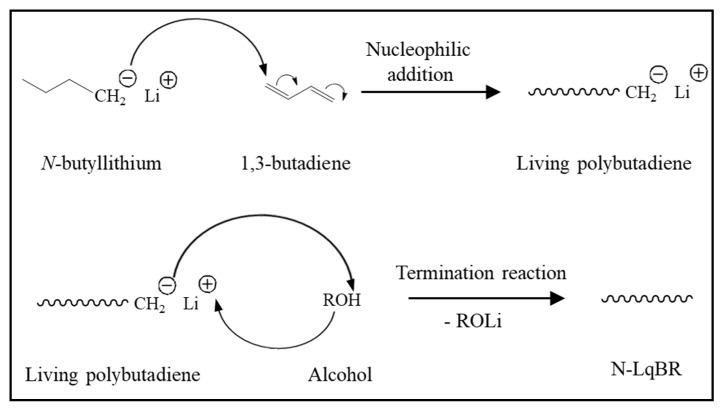
Polymerization of non-functionalized liquid butadiene rubber (N-LqBR).

**Scheme 2:**
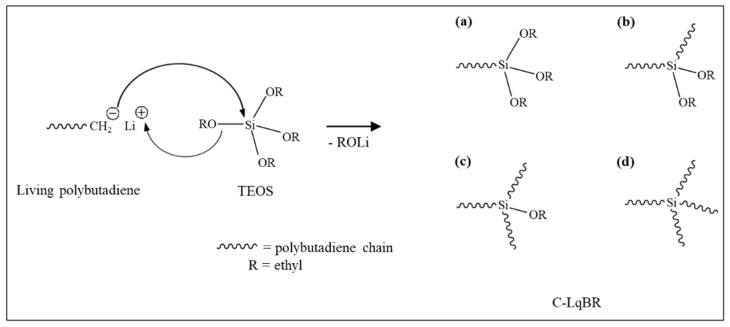
Polymerization of center-functionalized liquid butadiene rubber (C-LqBR); (**a**) BRSi(OR)_3_, (**b**) BR_2_Si(OR)_2_, (**c**) BR_3_Si(OR), and (**d**) BR_4_Si.

The formulation for N-LqBRs and C-LqBRs polymerization is shown in Table 1. Non-functionalized liquid BR (N-LqBR) and center-functionalized liquid BR (C-LqBR) were synthesized through anionic polymerization at 50 °C using a reactor purged with nitrogen. The amount of n-butyllithium was controlled to synthesize LqBR with different molecular weights, and anisole was inserted at a 6 molar ratio compared to n-butyllithium to have a low vinyl content [22]. Then, 1,3-butadiene was added to the reactor using nitrogen pressure. All the LqBRs were polymerized under the same reaction conditions and the reaction was terminated using benzyl alcohol (1.2 molar excess to the initiator) after reacting for 40 min (Scheme 1). In contrast, for C-LqBR, the reaction was terminated by adding TEOS (0.5 molar ratio) to synthesize it in a structure, wherein two BR chains are coupled (Scheme 2) [23,24]. Then, LqBR was obtained by evaporating cyclohexane in the LqBR solution using a vacuum evaporator. The macrostructure and microstructure of LqBR were analyzed using GPC and ^1^H NMR.

### 2.4. Preparation of SSBR/silica Compounds and Vulcanizates

The compound was prepared using an internal mixer (300cc, Mirae Scientific Instruments Inc., Gwangju, Korea) based on the formulation shown in Table 2. The fill factor was set to 70% of the mixer volume, and the input unit was parts per hundred rubber (phr). LqBR in the compound reduces the crosslink density by consuming sulfur and significantly influences the change in properties of vulcanizates [25,26,27,28]. A similar crosslink density is required to compare the properties of TDAE oil and LqBR-applied compounds. Therefore, the sulfur and CBS contents of the LqBR-applied compound were controlled to obtain a crosslink density similar to that of the TDAE oil-applied compound. The mixing procedure is as shown in Table 3, wherein the initial temperature of each stage was 100 and 50 °C, and the dump temperature was 150–155 °C and 80–90 °C, respectively. The compound was sheeted using a two-roll mill after mixing at each stage. The vulcanizates were prepared by pressing the compound in a hydraulic press at 160 °C for optimal curing time (t_90_).

## 3. Results and Discussion

### 3.1. Synthesis of LqBR

**Figure 1:**
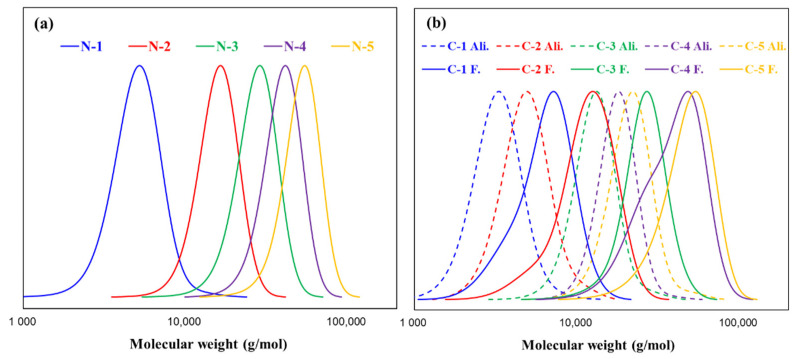
Gel permeation chromatography (GPC) curves of (**a**) N-LqBRs and (**b**) C-LqBRs.

**Figure 2:**
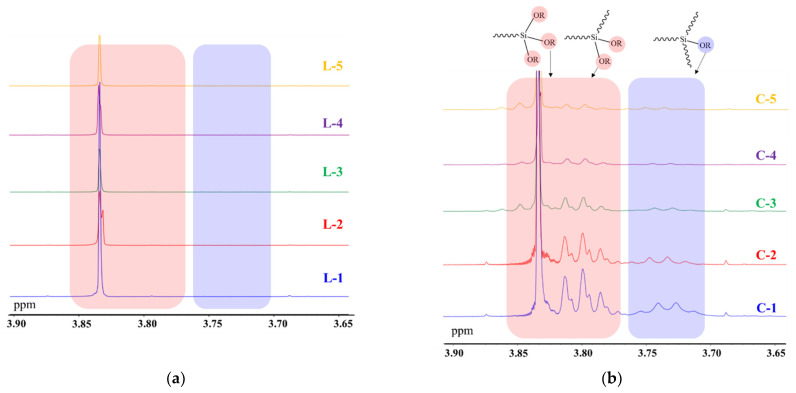
^1^H NMR spectra of (**a**) N-LqBRs and (**b**) C-LqBRs.

The GPC and ^1^H NMR analysis results of N-LqBRs and C-LqBRs are shown in Figure 1 and Figure 2 and Table 4. N-LqBRs and C-LqBRs have molecular weights in the range of 4800–50,000 g/mol and narrow molecular weight distributions (1.07–1.34) through the GPC results. Moreover, the 1,4-addition structure of butadiene peaked at 5.37–5.50 ppm and the 1,2-addition (vinyl) structure peaked at 4.79–4.99 and 5.50–5.60 ppm in the NMR spectra [29,30]. As a result of calculating the area ratio, the microstructures of all LqBRs (vinyl content: 13–15 wt%; 1,4-cis content: 45–47 wt%; 1,4-trans content: 38–42 wt%) were similarly controlled. The ^1^H chemical shift of the ^1^H in the alkoxy group (SiO-CH_2_-) varies based on the number of alkoxy groups bonded to the silicon atom [23,31]. The resonance peak shifts upfield as the number of alkoxy groups decreases. Therefore, resonance peaks were observed at 3.76–3.84 ppm for the tri and dialkoxysilyl groups and at 3.70–3.76 ppm for monoalkoxysilyl groups. The area of the ^1^H in the alkoxy group peak also decreased as the molecular weight of C-LqBR increased.

The coupling number (CN), which represents the number of coupled polybutadiene chains of C-LqBR, was calculated as the ratio of the number average molecular weight (M_n_) before and after the coupling reaction [32].

(4) $$\mathrm{Coupling}\;\mathrm{number}\;(\mathrm{CN})=\frac{\mathrm{Number}\;\mathrm{average}\;\mathrm{molecular}\;\mathrm{weight}\;\mathrm{after}\;\mathrm{coupling}}{\mathrm{Number}\;\mathrm{average}\;\mathrm{molecular}\;\mathrm{weight}\;\mathrm{before}\;\mathrm{coupling}}.$$

The CN of C-LqBR ranged from 1.9 to 2.3, indicating that the coupling reaction of two polybutadiene chains (Scheme 2b) was dominant.

### 3.2. Payne Effect

**Figure 3:**
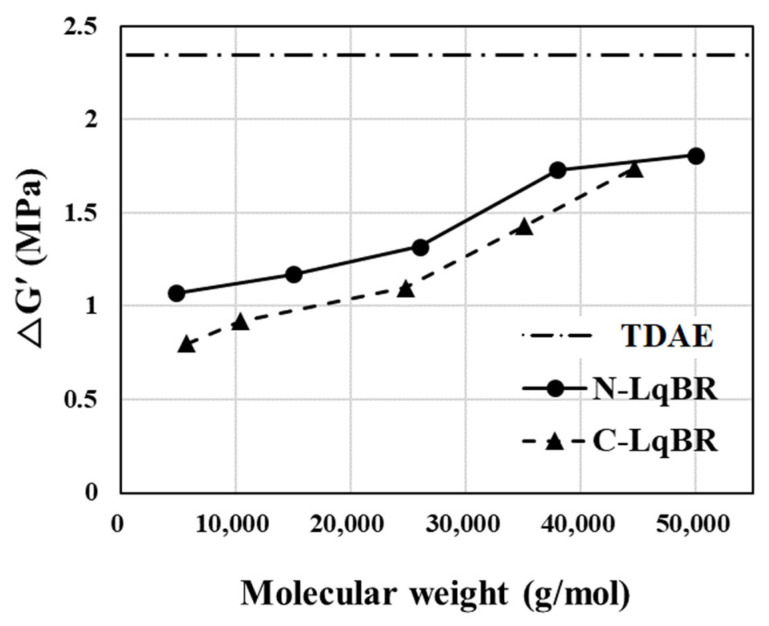
Variation in the △G′ values as a function of the molecular weight of LqBR.

The Payne effect, as shown in Figure 3 and Table 5, indicates the filler–filler interaction of the uncured compound, so indicating the degree of filler dispersion [33]. In addition, the decrease in storage modulus (G′) according to an increase in strain amplitude is a result of the destruction of the filler network, wherein a larger value of △G′ indicates a stronger filler–filler interaction.

**Figure 4:**
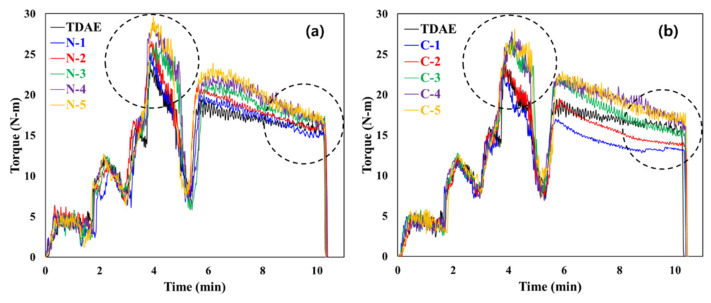
Mixing torque chart of the compounds; (**a**) N-LqBR compounds and (**b**) C-LqBR compounds.

Sufficient viscosity and shearing force are required during the mixing process for the outstanding silica dispersion of the compound. LqBR, which has a higher molecular weight than TDAE oil (molecular weight < 500 g/mol), has a high viscosity, thereby showing a high torque peak value during the mixing process, as shown in Figure 4. However, the final torque value became similar to that of TDAE because the mixing efficiency increased as the viscosity increased. Therefore, all the LqBR-applied compounds showed a lower Payne effect compared to TDAE, since silica dispersion was improved. In particular, C-LqBR showed a lower ΔG′ (0.28%–40.04% MPa) result compared to N-LqBR of similar molecular weight because silica dispersion was improved as the ethoxy group reacted with the silanol group on the silica surface. Moreover, the effect as a plasticizer was better when the molecular weight of LqBR was lower, and the ΔG′ value tended to be lower when there was a functional group.

### 3.3. Cure Characteristics and Mooney Viscosity of the Compounds

**Figure 5:**
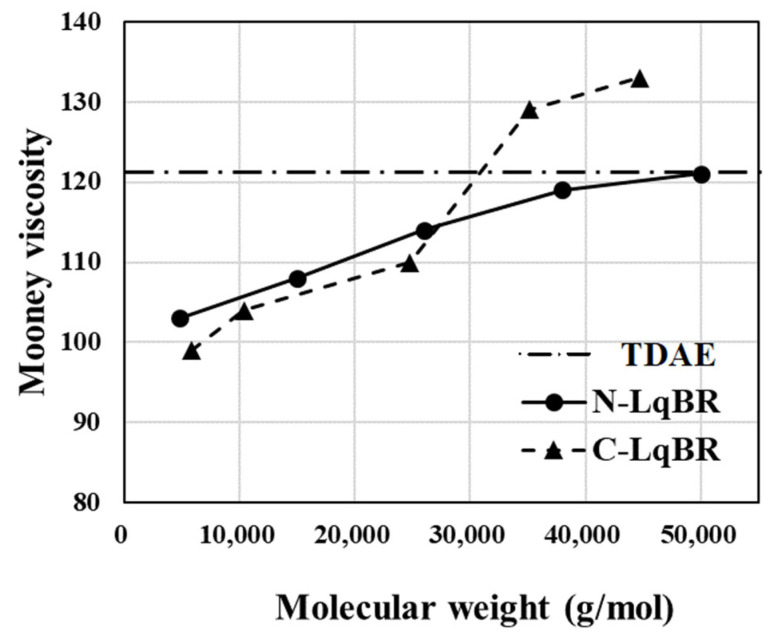
Mooney viscosity of the compounds as a function of the molecular weight of LqBR.

**Figure 6:**
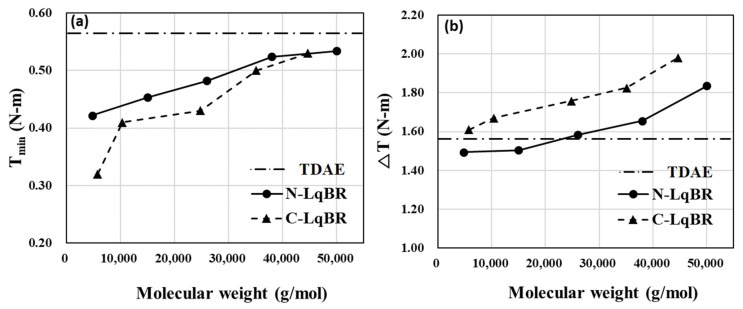
(**a**) T_min_ and (**b**) ΔT of the compounds as a function of the molecular weight of LqBR.

The results of Mooney viscosity related to the processability of the compound are shown in Figure 5 and Table 6. LqBR not only improved the silica dispersion but also showed a lower Mooney viscosity value than the TDAE compound because the chain slippage was facilitated by acting as a lubricant between the base polymer chains. In addition, the Mooney viscosity of the compound tended to increase due to the entanglement effect of the chain as the molecular weight of both N-LqBR and C-LqBR increased [34,35]. Although the C-LqBR compound showed a lower Mooney viscosity value than the N-LqBR compound because of the improved silica dispersion, the C-LqBR compound showed a higher viscosity value than the N-LqBR compound in the molecular weight range of 30,000 g/mol or above. This is because the filler-rubber interaction increased at the interface between the filler and polymer by C-LqBR as the molecular weight increased [36]. The results of cure characteristics obtained using a MDR are shown in Figure 6 and Table 6. The T_min_ value of the LqBR-applied compound was lower than that of the TDAE compound. This is a result of the improvement in the silica dispersion when LqBR was applied as in the Payne effect result. In addition, C-LqBR showed a lower T_min_ value compared to N-LqBR of similar molecular weight because the functional group can hydrophobize the silica surface through a silanization reaction.

In the case of silica compounds, the ΔT (T_max_ − T_min_) value in the MDR curve is known to be affected by the filler morphology and crosslink density [37]. In general, the ΔT values of the N-LqBR compounds are lower than that of TDAE because N-LqBR consumes sulfur which is planned to be used in the crosslink reaction between base polymers. However, in this evaluation, the ΔT value was similar to that of TDAE, as the sulfur and accelerator contents in the LqBR compound were controlled. In contrast, in terms of the C-LqBR, not only can the functional group be fixed on the silica surface but a chemical bond can also be formed through a crosslink reaction with the base polymer, thereby increasing the filler-rubber interaction. Therefore, it was determined that the ΔT value was higher than that of N-LqBR compounds despite the outstanding silica dispersion. Therefore, the crosslink density values of the C-LqBR compounds are expected to be higher than those of N-LqBR compounds [38].

### 3.4. Crosslink Density and Solvent Extraction

**Figure 7:**
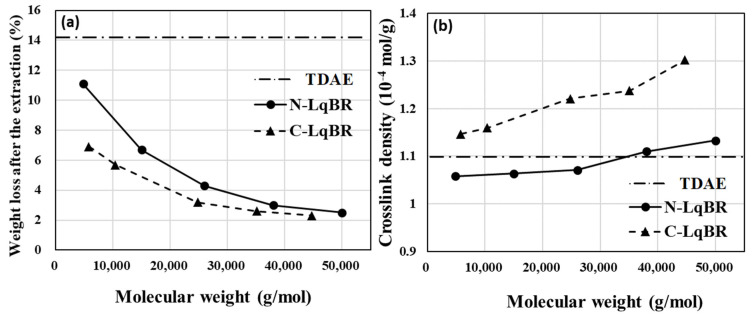
(**a**) Weight loss after the extraction and (**b**) crosslink density of the vulcanizates.

Organic matter was extracted from vulcanizate specimens using two types of organic solvents to determine the amount of organic matter in the vulcanizates. First, the oil and low molecular weight components that were added during mixing were extracted by THF. Then, soluble free LqBR was extracted from the specimen obtained after THF extraction using n-hexane. The total amount of organic matter extracted from the two types of organic solvents is shown in Figure 7a and Table 7.

The TDAE compound showed the highest organic matter extraction amount at 14.2%, since oil can easily be extracted by organic solvents as it does not form a chemical bond with other materials in the compound. In contrast, N-LqBR and C-LqBR compounds showed lower extraction than the TDAE compound because they can be fixed to the polymer network through co-vulcanization with the base polymer during vulcanization. In addition, C-LqBR showed a smaller amount of extraction compared to N-LqBR because functional groups can be fixed to the silica surface through a silanization reaction. The extraction amounts of N-5 and C-5 were the lowest among all compounds. This is because it is more advantageous for the crosslinking reaction with the base polymer when the molecular weight of LqBR is larger [8].

The crosslink density of vulcanizates is shown in Figure 7b and Table 7. LqBR generally reduces the crosslink density of vulcanizate because it consumes sulfur for use in base polymer crosslinking. The contents of sulfur and accelerator were controlled to accurately compare the physical properties of TDAE and LqBR compounds, and the N-LqBR compounds showed similar crosslink densities compared to the TDAE compound as a result of the swelling test. In addition, the C-LqBR compounds showed higher crosslink density values compared to the N-LqBR compounds due to the formation of a chemical bond between silica and rubber.

### 3.5. Mechanical Properties and DIN Abrasion Loss

**Figure 8:**
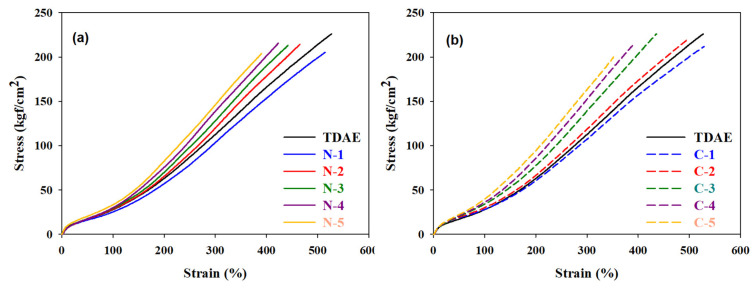
Stress–strain curves of the vulcanizates according to the applied (**a**) N-LqBRs and (**b**) C-LqBRs.

The modulus value in the stress–strain curves had a high correlation with the crosslink density result, as shown in Figure 8. The modulus 300 (M_300_) value increased because the crosslink density increased along with an increase in the molecular weight of LqBR, and C-LqBR, which is capable of the coupling reaction, had a higher M_300_ value than N-LqBR.

LqBR generally acts as a lubricant between the base polymer chains to facilitate slippage and decreases the modulus owing to a decrease in the crosslink density value by consuming sulfur. However, it showed a higher M_300_ value than that of the TDAE compound in all compounds except N-1 and C-1, as the crosslink density was similar to that of the TDAE compound by controlling the sulfur and accelerator.

**Figure 9:**
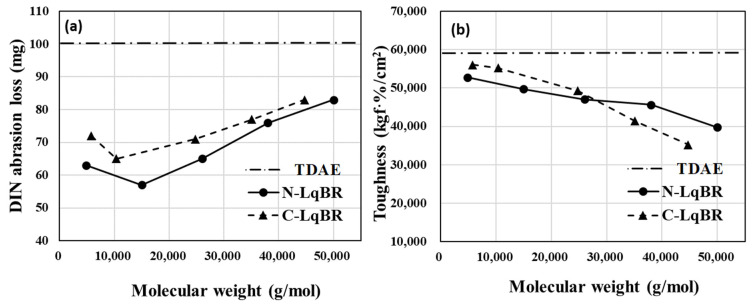
(**a**) Deutsche Industrie Normen (DIN) abrasion loss and (**b**) toughness of the vulcanizates according to the applied processing aids.

The mechanical properties and DIN abrasion loss measurement results are shown in Figure 8 and Figure 9 and Table 8. The abrasion resistance is significantly influenced by the T_g_, filler–rubber interaction, and toughness of the polymer [39,40,41,42]. LqBR, which has a lower T_g_ than TDAE oil, showed outstanding abrasion resistance by significantly lowering the T_g_ of the compound. The crosslink density increased with an increase in the molecular weight of LqBR, while the toughness calculated using the area of the stress–strain curves decreased. Thus, since the two factors affect abrasion resistance, the best abrasion resistance was confirmed in the N-2 and C-2 compounds.

### 3.6. Dynamic Viscoelastic Properties

**Figure 10:**
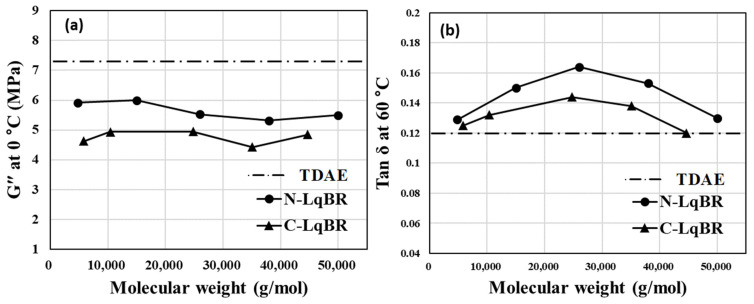
(**a**) G″ at 0 °C and (**b**) tan δ at 60 °C values of the vulcanizates according to the applied processing aids.

When the tire, to which the vehicle load is applied, rotates, energy loss occurs as all parts of the tire repeat deformation and recovery. This phenomenon is called hysteresis loss, which is the main cause of energy loss related to tire rolling resistance (RR). Moreover, during the braking, energy is lost as the tire is rapidly deformed on the road surface, which is related to the frictional force between the tire and the road surface. This hysteresis loss is attributable to the viscoelastic properties of the rubber compound and is measurable in the laboratory to predict tire performance. Among the viscoelastic properties, the storage modulus (G′) value in the low-temperature region below −10 °C is an indicator of snow traction and is known to improve snow traction at lower values [43,44]. This is because the tire tread can be deformed and adhered to the icy road surface more easily when the G′ value is lower under low-temperature conditions [45]. The loss modulus (G″) at 0 °C indicates the wet grip performance of the tire and is known to improve the wet grip performance when its value is higher [46,47]. In addition, the G″ value is higher when the effective filler volume fraction increases [48]. Tire RR can be calculated using tan δ at 60 °C, which is known to improve fuel efficiency when the value is lower [49]. Wang reported that tan δ at 60 °C characteristics are attributable to the destruction and reformation of the filler–filler network [48]. Kitamura reported that the hysteresis in this region are attributable to the free chain ends of the polymer [50]. Moreover, Salort reported that the hysteresis is caused by the dangling ends when low molecular weight butadiene rubber is crosslinked to the base polymer [8]. The results of both studies show that the free chain ends of the polymer are also an important factor in energy loss in the high-temperature region. The results of the dynamic viscoelastic properties of the compounds applied with TDAE oil and LqBR are shown in Table 9. All the LqBR compounds showed lower G′ values at −30 °C than TDAE compounds, regardless of the molecular weight. It was considered that LqBR with low T_g_ decreased the compound T_g_ and increased the flexibility of the compound at low temperatures. In the case of G″ at 0 °C, as shown in Figure 10a, all the LqBR compounds have lower values than TDAE compounds, which is inferred to be due to a significant decrease in T_g_ of vulcanizates. C-LqBR compounds are determined to have shown lower G″ at 0 °C than N-LqBR because the effective filler volume fraction was decreased as the silica dispersion was improved, similar to the Payne effect results earlier.

The tan δ value at 60 °C increased and then decreased as the molecular weight of N-LqBR and C-LqBR increased, as shown in Figure 10b. Since the molecular weights of N-1 and C-1 are lower than that between crosslink points M_c_ in vulcanizate, most of the LqBR chains are in a state where they cannot be bonded to the base polymer. However, the ratio of LqBR chains that can be bonded to the base polymer gradually increases as the molecular weight of LqBR increases, and this dangling chain causes hysteresis by behaving as free chain ends. In contrast, when most LqBRs reach the molecular weight range that can be bonded to the base polymer, such as N-3 and C-3, it is determined that the effect of decreasing the number of chains and chain end is more dominant than the dangling end effect. Therefore, tan δ at 60 °C tended to decrease due to a decrease in the number of LqBR chains from the molecular weight range beyond N-3 and C-3. In the case of C-LqBR, the value of tan δ at 60 °C was lower than that of the N-LqBR compound, which was attributed to the improvement of silica dispersion and the decrease in chain mobility due to the coupling reaction.

## 4. Conclusions

This study examined the effect of the molecular weight and modification of LqBR applied as a processing aid to silica-filled SSBR compounds on physical properties. Moreover, the properties were compared after controlling the sulfur and accelerator contents to have a crosslink density similar to that of the TDAE oil-applied compound. LqBR improved the Payne effect and processability without deteriorating the mechanical properties. In particular, C-LqBR with a functional group showed a lower Payne effect and Mooney viscosity compared to N-LqBR. N-LqBR compounds showed lower ΔT values than the TDAE compound by consuming sulfur during vulcanization, whereas C-LqBR showed a higher ΔT value as the functional group not only was fixed to the silica surface but also formed a chemical bond with the base polymer through a crosslink reaction. As the molecular weight of LqBR increased, the amount of organic matter extracted was reduced because it was more advantageous to the crosslink reaction with the base polymer. In terms of the mechanical properties, the modulus increased and the toughness decreased as the molecular weight of LqBR increased. The abrasion resistance showed optimum points in the N-2 and C-2 compounds. In particular, it was determined that not only the increase in the filler-rubber interaction but also the increase in toughness affected abrasion resistance. In terms of the dynamic properties, LqBR significantly lowered the T_g_ of the compound, thereby improving the snow traction. In the case of tan δ at 60 °C, the maximum value was observed because of both effects of the dangling ends from co-vulcanization and the decrease in the number of chains with the increase in molecular weight.

These characteristics of LqBR are suitable for winter tires that require high traction and long-term flexibility on the ice, and the results presented in this study are expected to provide the basis for optimal LqBR design and selection required for manufacturing LqBR-applied tires.

## Figures and Tables

**Table 1:** Formulation for N-LqBRs and C-LqBRs polymerization.

Processing Aid	N-LqBR					C-LqBR				
Sample Code	N1	N2	N3	N4	N5	C1	C2	C3	C4	C5
Butadiene (g)	60									
Cyclohexane (g)	300									
n-Butyllithium (mmol)	10.9	3.9	2.4	1.6	1.2	17.1	11.4	3.6	3.0	2.1
Anisole (mmol)	32.2	13.8	7.4	5.3	3.7	46.0	27.6	13.8	9.2	6.2
TEOS (mmol)	-	-	-	-	-	9.1	5.5	1.8	1.5	1.1
Benzyl alcohol (mmol)	12.2	4.6	3.0	2.3	1.5	-	-	-	-	-

**Table 2:** Formulation of the SSBR/silica compounds using processing aids (unit: phr).

Processing Aid	Treated Distillate Aromatic Extract (TDAE) Oil	N-LqBR					C-LqBR				
Sample Code	TDAE	N1	N2	N3	N4	N5	C1	C2	C3	C4	C5
SSBR	100	100									
Silica	100	100									
TESPT *	8	8									
TDAE oil	30	0	0	0	0	0	0	0	0	0	0
N-LqBR (No. 1–5)	0	30	30	30	30	30	0	0	0	0	0
C-LqBR (No. 1–5)	0	0	0	0	0	0	30	30	30	30	30
ZnO	3	3									
Stearic acid	2	2									
6PPD	1	1									
DPG	2.1	2.1									
Sulfur	0.7	1.15									
CBS	2.2	2.65									
ZBEC	0.2	0.2									

* The amounts of silane coupling agents are calculated as 8 wt% of the weight of silica.

**Table 3:** Mixing procedures.

	Time, min:s	Action
1st stage	0:00–0:40	Rubber (initial temp.: 100 °C)
	0:40–1:40	Silica 1/2 + silane1/2 + DPG1/2 + oil1/2
	1:40–2:40	Silica 1/2 + silane1/2 + DPG1/2 + oil1/2
	2:40–5:00	ZnO, St/A, 6PPD
	5:00	Ram up
	5:00–7:40	Extra mixing and dump (dump temp.: 150–155 °C)
2nd stage	0:00–0:20	Master batch from 1st stage (initial temp.: 50 °C)
	0:20–2:00	Curatives and dump (dump temp.: 80–90 °C)

**Table 4:** Characteristics of N-LqBRs and C-LqBRs.

Sample Code	Aliquot Sample ^a^ Mn (g/mol)	Final Sample ^b^ Mn (g/mol)	Polydispersity Index (PDI)	Vinyl Content (% in BD)	1,4-cis/trans (wt% in BD)	T_g_ (°C)	Coupling Number (CN)
N1	4800		1.14	14	45/41	−97	N/A
N2	15,000		1.09	13	45/42	−98	
N3	26,000		1.08	13	46/41	−97	
N4	38,000		1.08	14	45/41	−95	
N5	50,000		1.07	13	46/41	−96	
C1	3000	5800	1.18	15	46/39	−97	1.9
C2	4600	10,400	1.19	15	47/38	−96	2.3
C3	12,300	24,800	1.11	14	46/40	−94	2.0
C4	17,000	35,100	1.20	14	45/41	−95	2.1
C5	20,300	44,700	1.34	14	46/40	−95	2.2

^a^ Aliquot sample (Ali.); C-LqBR sample before reaction with TEOS. ^b^ Final sample (F.); C-LqBR sample after reaction with TEOS.

**Table 5:** The ΔG′ values according to the processing aids applied to the compounds.

Compound	TDAE	N1	N2	N3	N4	N5	C1	C2	C3	C4	C5
ΔG′ (0.28–40.04%, MPa)	2.34	1.07	1.17	1.32	1.73	1.81	0.8	0.92	1.1	1.43	1.74

**Table 6:** Cure characteristics and Mooney viscosity of the compounds.

Compound	Mooney Viscosity (ML_1+4_@100 °C)	T_min_ (N·m)	T_max_ (N·m)	ΔT (N·m)	t_10_ (min:s)	t_90_ _(_min:s)
TDAE	121	0.57	2.14	1.57	2:32	6:22
N1	103	0.42	1.92	1.50	2:20	4:59
N2	108	0.45	1.96	1.50	2:20	4:59
N3	114	0.48	2.07	1.58	2:08	4:52
N4	119	0.52	2.18	1.66	2:17	5:10
N5	121	0.53	2.37	1.84	2:23	4:59
C1	99	0.32	1.93	1.61	2:36	5:16
C2	104	0.41	2.08	1.67	2:26	5:08
C3	110	0.43	2.19	1.76	2:26	4:52
C4	129	0.50	2.33	1.83	2:25	4:47
C5	133	0.53	2.51	1.98	2:26	4:39

**Table 7:** Weight loss of the vulcanizates after the extraction and crosslink density.

Compound	TDAE	N1	N2	N3	N4	N5	C1	C2	C3	C4	C5
Crosslink density (10^−4^ mol/g)	1.10	1.05	1.06	1.07	1.11	1.13	1.15	1.16	1.22	1.24	1.30
Weight loss after the extraction (wt%)	14.2	11.1	6.7	4.3	3.0	2.5	6.9	5.7	3.2	2.6	2.3

**Table 8:** Mechanical properties and DIN abrasion loss of the vulcanizates.

Compound	M_100_(kgf/cm^2^)	M_300_(kgf/cm^2^)	Elongation at Break (%)	Tensile Strength (kgf/cm^2^)	Toughness (kgf/cm^2^)	Compound T_g_ (°C)	DIN Abrasion Loss (mg)
TDAE	27.5	112	527	226	59,551	−34.8	100
N1	25.0	103	515	205	52,788	−44.9	63
N2	28.1	120	465	214	49,755	−44.0	57
N3	29.2	127	442	213	47,073	−43.2	65
N4	29.9	139	423	216	45,631	−41.9	76
N5	33.4	146	390	204	39,805	−42.4	83
C1	28	107	529	212	56,074	−42.1	72
C2	30.1	118	502	220	55,220	−42.8	65
C3	33.8	139	436	226	49,313	−43.1	71
C4	35.7	152	389	213	41,429	−42.6	77
C5	39.5	163	352	200	35,200	−42.9	83

**Table 9:** Viscoelastic properties of the compounds according to the applied processing aids.

Compound	Compound T_g_ (°C)	G′ at −30 °C (MPa)	G″ at 0 °C (MPa)	Tan δ at 60 °C
TDAE	−34.8	140	7.06	0.120
N1	−44.9	75	5.92	0.129
N2	−44.0	77	6.00	0.150
N3	−43.2	76	5.53	0.164
N4	−41.9	80	5.32	0.153
N5	−42.4	86	5.50	0.130
C1	−42.1	63	4.63	0.125
C2	−42.8	66	4.94	0.132
C3	−43.1	67	4.95	0.144
C4	−42.6	64	4.43	0.138
C5	−42.9	72	4.85	0.120

## Data Availability

Data presented in this study are available on request from the corresponding author.
